# Relevance of Cor Pulmonale in COPD With and Without Pulmonary Hypertension: A Retrospective Cohort Study

**DOI:** 10.3389/fcvm.2022.826369

**Published:** 2022-02-16

**Authors:** Athiththan Yogeswaran, Stefan Kuhnert, Henning Gall, Marlene Faber, Ekaterina Krauss, Zvonimir A. Rako, Stanislav Keranov, Friedrich Grimminger, Hossein Ardeschir Ghofrani, Robert Naeije, Werner Seeger, Manuel J. Richter, Khodr Tello

**Affiliations:** ^1^Department of Internal Medicine, Member of the German Center for Lung Research, Universities of Giessen and Marburg Lung Center, Justus-Liebig-University Giessen, Giessen, Germany; ^2^Department of Internal Medicine, Member of the German Center for Lung Research, Institute for Lung Health, Cardio-Pulmonary Institute, Universities of Giessen and Marburg Lung Center, Giessen, Germany; ^3^Department of Cardiology and Angiology, DZHK (German Center for Cardiovascular Research), University of Giessen, Giessen, Germany; ^4^Department of Pathophysiology, Faculty of Medicine, Free University of Brussels, Brussels, Belgium

**Keywords:** chronic obstructive pulmonary disease, cor pulmonale, pulmonary arterial hypertension, right ventricle, risk stratification

## Abstract

**Background:**

The relevance of cor pulmonale in COPD and pulmonary hypertension due to COPD (PH-COPD) is incompletely understood. We aimed to investigate the relationship of right ventricular-pulmonary arterial (RV-PA) uncoupling with disease severity in COPD, and the relationship of RV-PA uncoupling and use of targeted PH therapies with mortality in PH-COPD.

**Methods:**

We retrospectively analyzed 231 patients with COPD without PH and 274 patients with PH-COPD. COPD was classified according to GOLD stages and the modified Medical Research Council dyspnoea scale. PH was categorized as mild-to-moderate or severe. RV-PA uncoupling was assessed as the echocardiographic tricuspid annular plane systolic excursion/pulmonary artery systolic pressure (TAPSE/PASP) ratio.

**Results:**

Of the cohort with COPD without PH, 21, 58, 54 and 92 were classified as GOLD I, II, III and IV, respectively. Patients in advanced GOLD stages and those with severe dyspnoea showed significantly decreased TAPSE/PASP.

Of the PH-COPD cohort, 144 had mild-to-moderate PH and 130 had severe PH. During follow-up, 126 patients died. In univariate Cox regression, TAPSE/PASP and 6-min walk distance (6MWD; 10 m increments) predicted survival [hazard ratios (95% CI): 0.12 (0.03–0.57) and 0.95 (0.93–0.97), respectively]; notably, PH severity and simplified European Society of Cardiology/European Respiratory Society risk stratification did not. Among patients in the lowest or intermediate tertiles of TAPSE/PASP and 6MWD, those with targeted PH therapy had higher survival than those without (53 vs. 17% at 3 years).

**Conclusion:**

Cor pulmonale (decreased TAPSE/PASP and 6MWD) is associated with disease severity in COPD and predicts outcome in PH-COPD.

## Introduction

Pulmonary hypertension (PH) as a complication of COPD is generally mild to moderate but can be severe in some patients (1). Mean pulmonary artery pressures (mPAP) higher than 35–40 mm Hg have been reported in 1–5% of patients with advanced COPD (2–4). PH has long been known to be associated with a reduced life expectancy in COPD, in proportion to increased PAP (5). Early studies also showed that PH due to COPD (PH-COPD) is associated with structural changes in the right ventricle or “cor pulmonale” (6). Altered right ventricular (RV) function was demonstrated by radionuclide angiography and clinicians learned that eventual systemic congestion symptomatology or “pulmonary heart disease” also heralded an increase in mortality in COPD (7–9). More recently, a validated echocardiographic measure of RV-pulmonary arterial (PA) coupling—the tricuspid annular plane systolic excursion (TAPSE)/pulmonary artery systolic pressure (PASP) ratio (10, 11)—was shown to be a strong predictor of outcome in PH on a background of either interstitial lung disease or COPD (12), as well as in heart failure (10, 13) and pulmonary arterial hypertension (PAH) (14). However, the extent to which RV dysfunction explains the altered functional state, decreased exercise capacity and decreased survival of patients with COPD is not exactly known. A risk scoring system for PAH proposed by the European Society of Cardiology and the European Respiratory Society (ESC/ERS) (15) has been successfully transposed to patients with PH due to interstitial lung disease (16) but its utility in patients with PH-COPD remains unknown. Whether targeted PH therapies (which have shown efficacy in PAH) might improve outcome in PH-COPD also remains undecided (1).

We therefore aimed to assess the relationship of disease severity with RV function in patients with COPD without PH, and the relationship of mortality with RV function, PAH-based risk scores and use of targeted PH therapies in patients with PH-COPD.

## Methods

### Patients and Study Design

We performed a two-part retrospective cohort study. In the first part, we included 231 patients with COPD without PH who had TAPSE and PASP data available. Echocardiographic and lung function parameters, 6-min walk distance (6MWD), Global Initiative for Obstructive Lung Disease (GOLD) stage, COPD Assessment Test (CAT), and modified Medical Research Council (mMRC) dyspnoea score were evaluated during routine visits to the Department of Pneumology in the Universities of Giessen and Marburg Lung Center. Routine visits took place between 4 August 2010 and 16 July 2021.

In the second part of the study, we included 274 patients with PH-COPD who were enrolled in the Giessen PH registry (17) between August 1995 and December 2018 and who had not previously received targeted PH therapy; some of these patients had also been included in a previously published study (12). Right heart catheterization was performed by experts, with haemodynamic measurements assessed after a short resting period (18). All enrolled patients were followed until June 2020. Survival status was determined by contacting the patient or their physician. Use of PH-specific drugs in severe PH-COPD was decided by experts based on assessment of the individual benefit-risk ratio, as recommended in the current guidelines (15).

For patients who were diagnosed with COPD in another centre, the baseline parameters of lung function are missing due to lack of access.

All patients gave written informed consent. The study was approved by the University of Giessen institutional review board (#266/11).

### Haemodynamic Classification of PH-COPD

The date of the initial right heart catheterization was taken as the date of PH diagnosis. The final diagnosis was made by a multidisciplinary board including physicians, radiologists and surgeons. PH-COPD was classified as mild-to-moderate or severe according to mPAP and cardiac index as recommended by an expert working group at the 6th World Symposium on PH (1): mPAP between 25 and 34 mm Hg alone or mPAP between 21 and 24 mm Hg with pulmonary vascular resistance (PVR) ≥3 Wood Units was classified as mild-to-moderate PH-COPD, and mPAP ≥35 mm Hg alone or mPAP ≥25 mm Hg with cardiac index <2.0 L/min/m^2^ was classified as severe PH-COPD.

### Risk Stratification in PH-COPD

Risk assessment in PH-COPD was performed using a validated simplified version (19) of the ESC/ERS risk stratification system (15). In brief, patients were categorized into low-, intermediate- and high-risk groups based on 6MWD, brain natriuretic peptide (BNP), right atrial pressure (RAP), cardiac index, mixed venous oxygen saturation (SvO_2_), right atrial area (echocardiography), World Health Organization functional class and the presence of pericardial effusion (echocardiography), all according to the cut-offs mentioned in the ESC/ERS guidelines (15).

RV-PA coupling was assessed using the TAPSE/PASP ratio determined by echocardiography.

### Statistical Analyses

Baseline characteristics are shown as mean ± standard deviation if normally distributed and as median [interquartile range (IQR)] if non-normally distributed. Comparisons between subgroups were performed using either Student's *t*-tests or non-parametric tests. Descriptive statistics and correlation analyses were used to evaluate the importance of the TAPSE/PASP ratio in COPD without PH.

In patients with PH-COPD, univariate Cox regression analysis was performed including age, PVR, BNP, 6MWD, SvO_2_, RAP, cardiac index, mPAP, right atrial area, the TAPSE/PASP ratio, forced vital capacity (FVC), total lung capacity (TLC), forced expiratory volume in 1 second (FEV_1_), the FEV_1_/vital capacity (VC) ratio, lung diffusing capacity for carbon monoxide (DLCO), PH-COPD severity (mild-to-moderate or severe), ESC/ERS risk score and body mass index, obstruction, dyspnoea and exercise capacity (BODE) index. TAPSE or PASP alone were not added due to collinearity. All variables that showed a significant association with mortality were included in a multivariate, stepwise, backward Cox regression model to identify independent predictors of mortality in patients with PH-COPD. Cut-off values with the highest sensitivity and specificity for predicting mortality were identified by receiver operating characteristic analysis and calculation of Youden's index. No imputation for missing data was implemented. Survival analyses were conducted using Kaplan–Meier plots (truncated at 5 years) and log-rank tests.

All analyses were performed using R 4.0 (the R Foundation, Vienna, Austria) and SPSS 26.0 (IBM, Armonk, USA). In the stepwise backward model, parameters with *p* > 0.1 were excluded. For all other analyses, *p* < 0.05 was considered significant.

## Results

### Study Population With COPD

Baseline characteristics of the 231 patients with COPD without PH are shown in Table 1. The median (IQR) age was 66 (59, 72) years and 48% of the patients were female. FEV_1_/VC ratio, FEV_1_, TLC and FVC data were available in 226 (98%), 226 (98%), 223 (97%), and 223 (97%) patients, respectively. BNP concentration, 6MWD, CAT score and mMRC score were available in 209 (90%), 73 (32%), 179 (77%) and 119 (52%) patients, respectively. The TAPSE/PASP ratio did not differ between male and female patients (*p* = 0.84).

**Table 1:** Baseline characteristics of patients with COPD without pulmonary hypertension.

	**GOLD stage**				
	**I (*n* = 21)**	**II (*n* = 58)**	**III (*n* = 54)**	**IV (*n* = 92)**	**Unknown (*n* = 6)**
Age, years	61.0 [56.0, 74.0]	67.0 [60.0, 72.8]	65.0 [60.0, 71.5]	66.0 [59.0, 71.0]	60.5 [51.3, 72.0]
*n* with data	21	58	54	92	6
Male sex, *n* (%)	9 (43)	27 (47)	32 (59)	47 (51)	4 (67)
*n* with data	21	58	54	92	6
Pack years	40.0 [30.0, 55.0]	44.5 [30.0, 50.0]	45.0 [30.0, 60.0]	40.0 [30.0, 50.0]	50.0 [35.5, 52.5]
*n* with data	19	52	49	73	3
BMI, kg/m^2^	26.4 [24.2, 29.7]	26.8 [23.5, 30.7]	26.1 [23.5, 28.9]	23.2 [20.4, 28.9]	25.0 [23.1, 26.7]
*n* with data	20	58	54	92	5
FEV_1_, % pred	81.8 [75.6, 87.2]	60.8 [54.0, 69.1]	40.4 [34.7, 44.8]	26.7 [22.6, 32.8]	61.4 [29.7, 98.5]
*n* with data	21	58	52	91	4
FEV_1_/VC max	66.2 [64.1, 71.0]	55.7 [47.3, 62.3]	45.3 [38.2, 49.9]	36.2 [30.9, 44.9]	50.0 [33.8, 70.5]
*n* with data	21	58	52	91	4
TLC, % pred	115 [108, 121]	114 [100, 123]	118 [102, 127]	125 [112, 140]	114 [103, 124]
*n* with data	21	57	51	90	4
VC, % pred	98.4 [93.2, 106]	89.7 [76.2, 99.3]	72.8 [63.1, 82.0]	60.0 [49.1, 70.9]	78.3 [67.2, 99.9]
*n* with data	21	57	51	90	4
FVC, % pred	90.3 [81.1, 104]	78.4 [69.9, 91.5]	61.2 [54.8, 73.7]	52.0 [43.8, 60.7]	72.8 [60.0, 97.2]
*n* with data	20	57	52	90	4
DLCO, % pred	62.7 [50.7, 70.8]	45.5 [36.7, 69.3]	36.8 [29.5, 42.6]	22.4 [17.4, 29.4]	47.1 [45.2, 59.0]
*n* with data	20	52	43	46	3
TAPSE, mm	23.0 [21.0, 27.0]	22.5 [20.0, 25.0]	22.0 [20.3, 24.8]	22.0 [19.8, 25.0]	23.5 [22.0, 25.0]
*n* with data	21	58	54	92	6
PASP, mm Hg	24.0 [21.0, 30.0]	30.0 [25.0, 36.8]	34.5 [27.0, 39.0]	35.5 [30.0, 45.0]	33.5 [28.5, 34.0]
*n* with data	21	58	54	92	6
TAPSE/PASP ratio, mm/mm Hg	0.90 [0.75, 1.04]	0.76 [0.59, 0.94]	0.68 [0.57, 0.82]	0.60 [0.47, 0.76]	0.76 [0.68, 0.80]
*n* with data	21	58	54	92	6
BNP, pg/mL	24.5 [13.8, 58.5]	30.0 [15.3, 63.8]	22.0 [12.0, 52.3]	37.0 [18.0, 73.3]	24.0 [10.0, 89.0]
*n* with data	16	54	52	82	5
Creatinine, mg/dL	0.75 [0.70, 1.03]	0.80 [0.70, 1.00]	0.80 [0.70, 0.90]	0.80 [0.63, 1.00]	0.95 [0.83, 1.08]
*n* with data	16	55	54	90	6
6MWD, m	411 ± 145	365 ± 80.4	353 ± 91.6	257 ± 121	269 ± 148
*n* with data	5	13	14	39	2
**mMRC dyspnoea score**, ***n*** **(%)**					
0	1 (4.8)	5 (8.6)	3 (5.6)	0 (0)	1 (17)
1	3 (14)	7 (12)	5 (9.3)	2 (2.2)	0 (0)
2	2 (10)	11 (19)	6 (11)	3 (3.3)	0 (0)
3	2 (10)	11 (19)	18 (33)	18 (20)	0 (0)
4	0 (0)	0 (0)	5 (9.3)	16 (17.4)	0 (0)
CAT score	23.0 [15.5, 25.8]	22.0 [14.0, 25.0]	26.5 [19.5, 30.0]	26.0 [21.8, 30.0]	13.0 [9.50, 16.5]
*n* with data	18	53	46	60	2
O_2_ at rest (L/min)	3.00 [3.00, 3.00]	2.00 [2.00, 3.00]	2.00 [2.00, 2.00]	2.00 [2.00, 3.00]	3.00 [3.00, 3.00]
*n* with data^a^	2	12	18	70	1
**Long-acting beta-agonist**, ***n*** **(%)**					
Unknown	0 (0)	1 (1.7)	4 (7.4)	4 (4.3)	3 (50)
No	9 (43)	10 (17)	4 (7.4)	2 (2.2)	1 (17)
Yes	12 (57)	47 (81)	46 (85)	86 (93)	2 (33)
**Long-acting muscarinic antagonist**, ***n*** **(%)**					
Unknown	0 (0)	2 (3.4)	4 (7.4)	4 (4.3)	3 (50)
No	6 (29)	10 (17)	4 (7.4)	5 (5.4)	1 (17)
Yes	15 (71)	46 (79)	46 (85)	83 (90)	2 (33)
**Inhaled corticosteroids**, ***n*** **(%)**					
Unknown	0 (0)	2 (3.4)	6 (11)	4 (4.3)	4 (67)
No	12 (57)	27 (47)	10 (19)	9 (9.8)	0 (0)
Yes	9 (43)	29 (50)	38 (70)	79 (86)	2 (33)
**Theophylline**, ***n*** **(%)**					
Unknown	4 (19)	7 (12)	8 (15)	11 (12)	3 (50)
No	17 (81)	49 (84)	42 (78)	70 (76)	3 (50)
Yes	0 (0)	2 (3.4)	4 (7.4)	11 (12)	0 (0)
**Roflumilast**, ***n*** **(%)**					
Unknown	5 (24)	7 (12)	9 (17)	13 (14)	3 (50)
No	16 (76)	48 (83)	34 (63)	62 (67)	3 (50)
Yes	0 (0)	1 (1.7)	10 (19)	12 (13)	0 (0)
Discontinued	0 (0)	2 (3.4)	1 (1.9)	5 (5.4)	0 (0)

*Data are presented as median [interquartile range], mean ± standard deviation or n (%). Numbers of patients with available data are shown in italics. 6MWD, 6-min walk distance; % pred, % predicted; BMI, body mass index; BNP, brain natriuretic peptide; CAT, COPD Assessment Test; DLCO, lung diffusing capacity for carbon monoxide; FEV_1_, forced expiratory volume in 1 s; FVC, forced vital capacity; GOLD, Global Initiative for Obstructive Lung Disease; max, maximum; mMRC, modified Medical Research Council; O_2_, oxygen; PASP, pulmonary artery systolic pressure; TAPSE, tricuspid annular plane systolic excursion; TLC, total lung capacity; VC, vital capacity.*

a *A total of 103 patients received O_2_ supplementation; all other patients did not receive O_2_ supplementation*.

### Relevance of TAPSE/PASP in COPD

The TAPSE/PASP ratio differed between GOLD stages I–IV (ANOVA *p* < 0.001). Patients in higher GOLD stages showed significantly lower TAPSE/PASP ratios (Table 1). Concordantly, patients with severe dyspnoea (mMRC grade III or IV) exhibited a significantly decreased TAPSE/PASP ratio compared with patients with less severe symptoms [mMRC III/IV: 0.61 (0.50, 0.81) mm/mm Hg; mMRC I/II: 0.75 (0.60, 0.93) mm/mm Hg; *p* = 0.03], and patients who required oxygen supplementation showed a lower TAPSE/PASP ratio than those who did not [0.59 (0.42, 0.74) mm/mm Hg vs. 0.76 (0.62, 0.95) mm/mm Hg; *p* < 0.001]. Interestingly, patients with exacerbations leading to inpatient treatment also showed a significantly lower TAPSE/PASP ratio than those without such exacerbations [0.65 (0.48, 0.77) mm/mm Hg vs. 0.76 (0.60, 0.95) mm/mm Hg; *p* = 0.001]. Patients with a higher BODE index had a significantly lower TAPSE/PASP ratio (*p* = 0.034; Supplementary Figure 1A).

Correlations of the TAPSE/PASP ratio with different parameters including lung function are shown in Table 2. The TAPSE/PASP ratio showed meaningful correlations with age, 6MWD, FEV_1_ and DLCO.

**Table 2:** Correlations with the TAPSE/PASP ratio in patients with COPD without pulmonary hypertension.

**Parameter**	**Pearson R**	***P*-value**	**Spearman rho**	***P*-value**
Age, years	−0.316	<0.001	−0.297	<0.001
CAT score	−0.141	0.06	−0.133	0.08
Pack years	−0.153	0.03	−0.089	0.2
6MWD, m	0.314	0.007	0.326	0.005
FEV_1_, % pred	0.310	<0.001	0.340	<0.001
FEV_1_/VC max	0.235	<0.001	0.277	<0.001
TLC, % pred	−0.123	0.07	−0.092	0.2
VC, % pred	0.281	<0.001	0.274	<0.001
FVC, % pred	0.246	<0.001	0.254	<0.001
DLCO, % pred	0.423	<0.001	0.414	<0.001

*6MWD, 6-min walk distance; % pred, % predicted; CAT, COPD Assessment Test; DLCO, lung diffusing capacity for carbon monoxide; FEV_1_, forced expiratory volume in 1 s; FVC, forced vital capacity; max, maximum; PASP, pulmonary artery systolic pressure; TAPSE, tricuspid annular plane systolic excursion; TLC, total lung capacity; VC, vital capacity*.

### Study Population With PH-COPD

Baseline characteristics of the 274 included patients with PH-COPD are shown in Table 3. The median (IQR) age was 70 (65, 78) years, and most of the patients (62%) were male. The FEV_1_/VC ratio, FEV_1_ and FVC were reduced, PH was on average mild to moderate and the 6MWD was low. In total, 144 patients (53%) had mild-to-moderate PH-COPD and 130 patients (47%) had severe PH-COPD. Patients with severe PH-COPD had normal TLC, higher FEV_1_/VC ratios, FEV_1_ and FVC and more severe haemodynamic impairment than patients with mild-to-moderate PH-COPD (Table 3). The TAPSE/PASP ratio did not differ between male and female patients (*p* = 0.56), and was slightly but non-significantly lower in patients with a higher BODE index (*p* = 0.50; Supplementary Figure 1B).Twenty-one patients were lost to follow-up and were therefore excluded from survival analysis.

**Table 3:** Baseline characteristics of patients with PH due to COPD.

	**Mild-to-moderate PH due to COPD** **(*N* = 144)**	**Severe PH due to COPD** **(*N* = 130)**
Age, years	72.0 [65.0, 76.3]	69.0 [64.0, 78.0]
*n* with data	144	130
Male sex, *n* (%)	86 (60)	84 (65)
*n* with data	144	130
Body mass index, kg/m^2^	24.9 [21.5, 29.6]	24.3 [22.3, 27.6]
*n* with data	144	130
FEV_1_, % pred	39.0 [28.8, 55.7]	50.5 [32.2, 68.2]
*n* with data	137	128
FEV_1_/VC max	48.2 [40.2, 60.0]	55.2 [47.0, 65.5]
*n* with data	125	115
FVC, % pred	66.3 ± 21.6	72.2 ± 23.7
*n* with data	116	114
TLC, % pred	114 [99.0, 125]	105 [91.5, 117]
*n* with data	130	123
DLCO, % pred	40.2 [27.4, 50.7]	32.3 [24.6, 43.6]
*n* with data	71	80
mPAP, mm Hg	28.0 [26.0, 31.0]	40.0 [36.0, 46.0]
*n* with data	144	130
PVR, dyn·s/cm^5^	299 [248, 368]	542 [427, 699]
*n* with data	130	107
Cardiac index, L/min/m^2^	2.84 [2.45, 3.28]	2.40 [1.90, 2.86]
*n* with data	144	129
SvO_2_, %	68.8 [64.2, 71.9]	64.5 [58.7, 69.4]
*n* with data	143	128
RAP, mm Hg	4.00 [2.00, 6.00]	5.50 [3.00, 9.00]
*n* with data	143	128
BNP, pg/mL	55.0 [23.0, 128]	165 [57.0, 355]
*n* with data	122	110
6MWD, m	271 ± 102	241 ± 105
*n* with data	110	101
TAPSE/PASP ratio, mm/mm Hg	0.407 [0.309, 0.555]	0.286 [0.207, 0.356]
*n* with data	88	91
**Targeted PH therapy**, ***n*** **(%)**		
No	82 (57)	30 (23)
Yes	57 (40)	93 (72)
Combination therapy	3 (2.1)	4 (3.1)
Monotherapy	54 (38)	89 (68)
**Phosphodiesterase 5 inhibitor**		
No	2 (1.4)	17 (13)
Yes	55 (38)	76 (58)
**Endothelin receptor antagonist**		
No	54 (38)	75 (58)
Yes	3 (2.1)	18 (14)

*Data are presented as median [interquartile range], mean ± standard deviation or n (%). Numbers of patients with available data are shown in italics. 6MWD, 6-min walk distance; % pred, % predicted; BNP, brain natriuretic peptide; DLCO, lung diffusing capacity for carbon monoxide; FEV_1_, forced expiratory volume in 1 s; FVC, forced vital capacity; max, maximum; mPAP, mean pulmonary artery pressure; PASP, pulmonary artery systolic pressure; PH, pulmonary hypertension; PVR, pulmonary vascular resistance; RAP, right atrial pressure; SvO_2_, mixed venous oxygen saturation; TAPSE, tricuspid annular plane systolic excursion; TLC, total lung capacity; VC, vital capacity*.

### Predictors of Mortality in PH-COPD

During follow-up (truncated at 5 years after diagnosis), 126 patients died. Median survival was 53 months. In univariate analysis, mPAP, PVR, the TAPSE/PASP ratio, 6MWD, FVC and the BODE index significantly predicted mortality whereas age, PH-COPD severity, ESC/ERS risk score, BNP, right atrial area, SvO_2_, RAP, cardiac index, DLCO and other lung function tests did not (Table 4), although further analysis of the prognostic capability of DLCO revealed an association with short-term mortality (truncated at 2 years after diagnosis; Supplementary Figure 3). The TAPSE/PASP ratio correlated with mPAP, cardiac index and SvO_2_ (Supplementary Table 1). Only the TAPSE/PASP ratio and 6MWD independently predicted mortality (Table 4).

**Table 4:** Univariate and multivariate Cox regression survival analyses.

**Parameter**	**Univariate HR (95% CI)**	**Multivariate HR (95% CI)**
Age	0.98 (0.96–1.00)	–
PVR (per 100 dyn·s/cm^5^ increase)	1.10 (1.00–1.20)	1.06 (0.92–1.22)
TAPSE/PASP ratio	0.12 (0.03–0.57)	0.27 (0.04–1.94)^a^
BNP (per 100 pg/mL)	1.00 (0.97–1.10)	–
DLCO	0.99 (0.97–1.00)	–
FEV_1_/VC max (per 10 units increase)	1.00 (0.91–1.20)	–
FEV_1_ (per 10% pred increase)	0.96 (0.87–1.10)	–
FVC	0.99 (0.98–1.00)	0.99 (0.98–1.00)
TLC	0.99 (0.98–1.00)	–
6MWD (per 10 m increase)	0.95 (0.93–0.97)	0.95 (0.92–0.99)^a^
SvO_2_	0.98 (0.95–1.00)	–
RAP	1.00 (0.97–1.00)	–
Cardiac index	0.90 (0.69–1.20)	–
mPAP (per 5 mm Hg increase)	1.10 (1.00–1.20)	1.00 (0.80–1.30)
Right atrial area	1.00 (0.99–1.10)	–
**PH-COPD severity**		
Severe vs. mild-to-moderate	1.32 (0.88–1.99)	–
**ESC/ERS risk score**		
Intermediate vs. low	1.32 (0.91–1.93)	–
High vs. low	2.34 (0.83–6.59)	–
**BODE index^b^**		
High (>6) vs. intermediate (3–6)	0.526 (0.287–0.962)	–

*6MWD, 6-min walk distance; % pred, % predicted; BNP, brain natriuretic peptide; BODE, body mass index, obstruction, dyspnoea and exercise capacity; DLCO, lung diffusing capacity for carbon monoxide; ESC/ERS, European Society of Cardiology and European Respiratory Society; FEV_1_, forced expiratory volume in 1 s; FVC, forced vital capacity; HR, hazard ratio; mPAP, mean pulmonary artery pressure; PASP, pulmonary artery systolic pressure; PH-COPD, pulmonary hypertension due to COPD; PVR, pulmonary vascular resistance; RAP, right atrial pressure; SvO_2_, mixed venous oxygen saturation; TAPSE, tricuspid annular plane systolic excursion; TLC, total lung capacity; VC, vital capacity.*

a *Independently predicted mortality in a stepwise backward model [Step 4, HR (95% CI): TAPSE/PASP ratio, 0.22 (0.04–1.36); 6MWD per 10 m increase, 0.95 (0.92–0.98)].*

b *Kaplan–Meier analysis revealed significant differences in survival between the three BODE index groups (Supplementary Figure 2), but no HR was computable for patients with a low BODE index (≤ 2) due to the small sample size. The BODE index was not included in the multivariate Cox regression analysis owing to a high number of missing values*.

**Figure 1:**
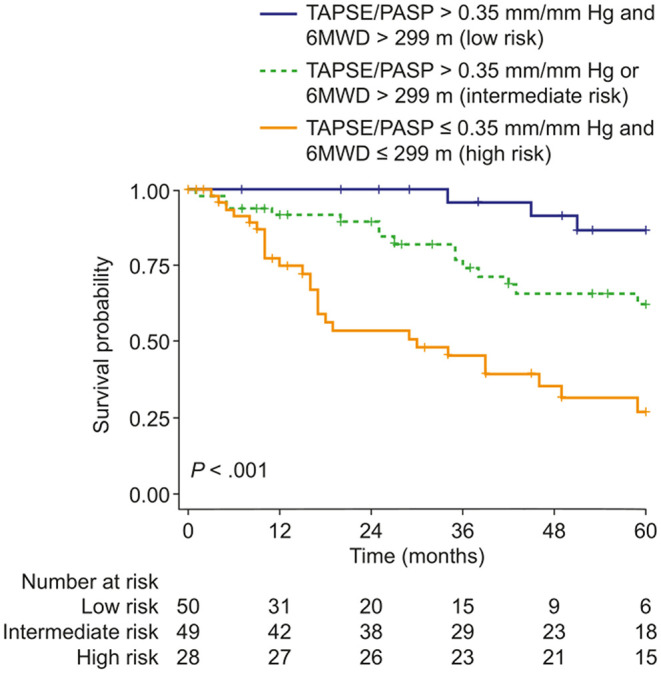
Kaplan–Meier curves of survival probability in patients with pulmonary hypertension due to COPD stratified according to the presence/absence of two risk factors: 6MWD ≤ 299 m and/or TAPSE/PASP ratio ≤ 0.35 mm/mm Hg (thresholds determined by receiver operating characteristic analysis and Youden's index). 6MWD, 6-min walk distance; TAPSE/PASP, tricuspid annular plane systolic excursion/pulmonary artery systolic pressure.

Prognostic cut-off values determined by Youden's index were 0.35 mm/mm Hg for the TAPSE/PASP ratio and 299 m for 6MWD. Patients were classified into three risk groups based on the two predictors: low risk (6MWD and TAPSE/PASP above the cut-off values), intermediate risk (6MWD or TAPSE/PASP above the cut-off value), and high risk (neither 6MWD nor TAPSE/PASP above the cut-off values). As illustrated in Figure 1, survival at 1, 3 and 5 years was 100, 96, and 87%, respectively, in the low-risk group, 92, 74, and 62%, respectively, in the intermediate-risk group, and 75, 46, and 27%, respectively, in the high-risk group. Cox regression revealed that the risk of mortality was increased 4-fold in the intermediate-risk group [hazard ratio (HR): 3.63; 95% CI: 1.05–12.6] and 11-fold in the high-risk group (HR: 10.5; 95% CI: 3.17–34.9).

**Figure 2:**
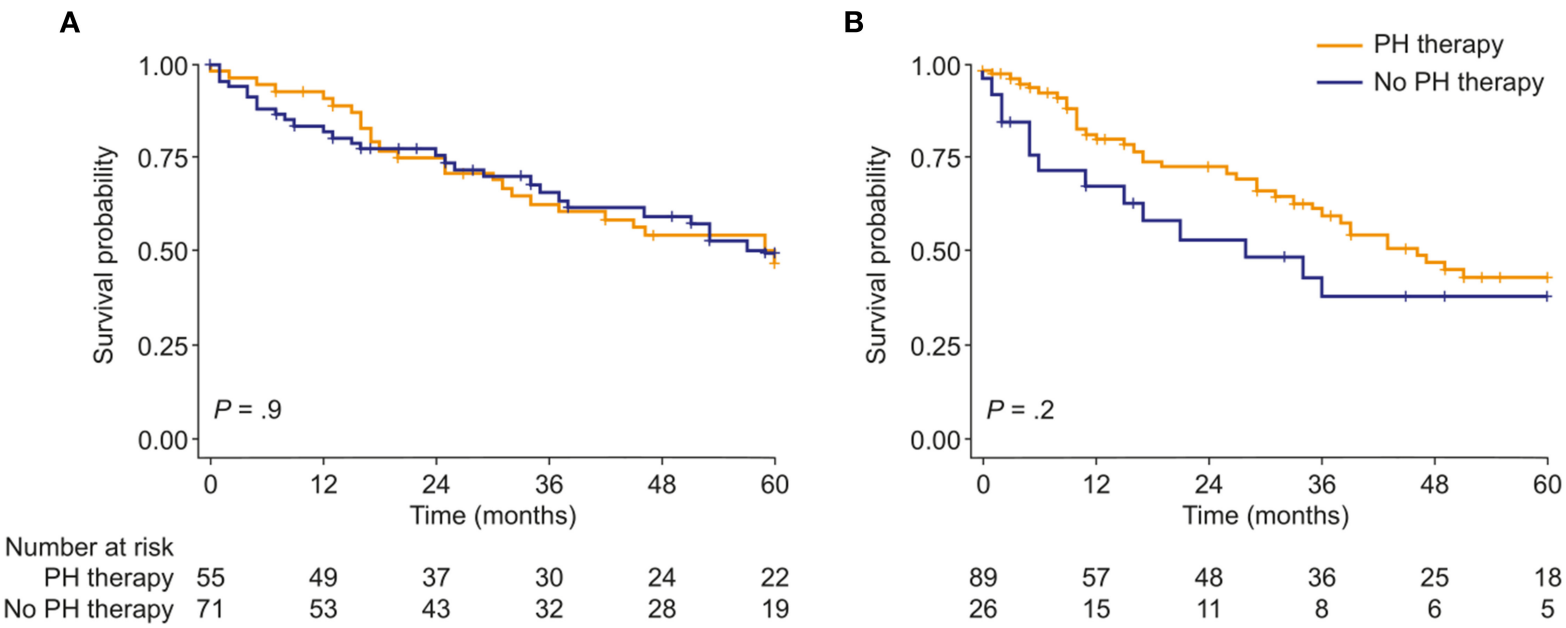
Kaplan–Meier curves of survival probability in patients with either **(A)**, mild-to-moderate or **(B)**, severe PH due to COPD, stratified by use of targeted PH therapy. PH, pulmonary hypertension.

**Figure 3:**
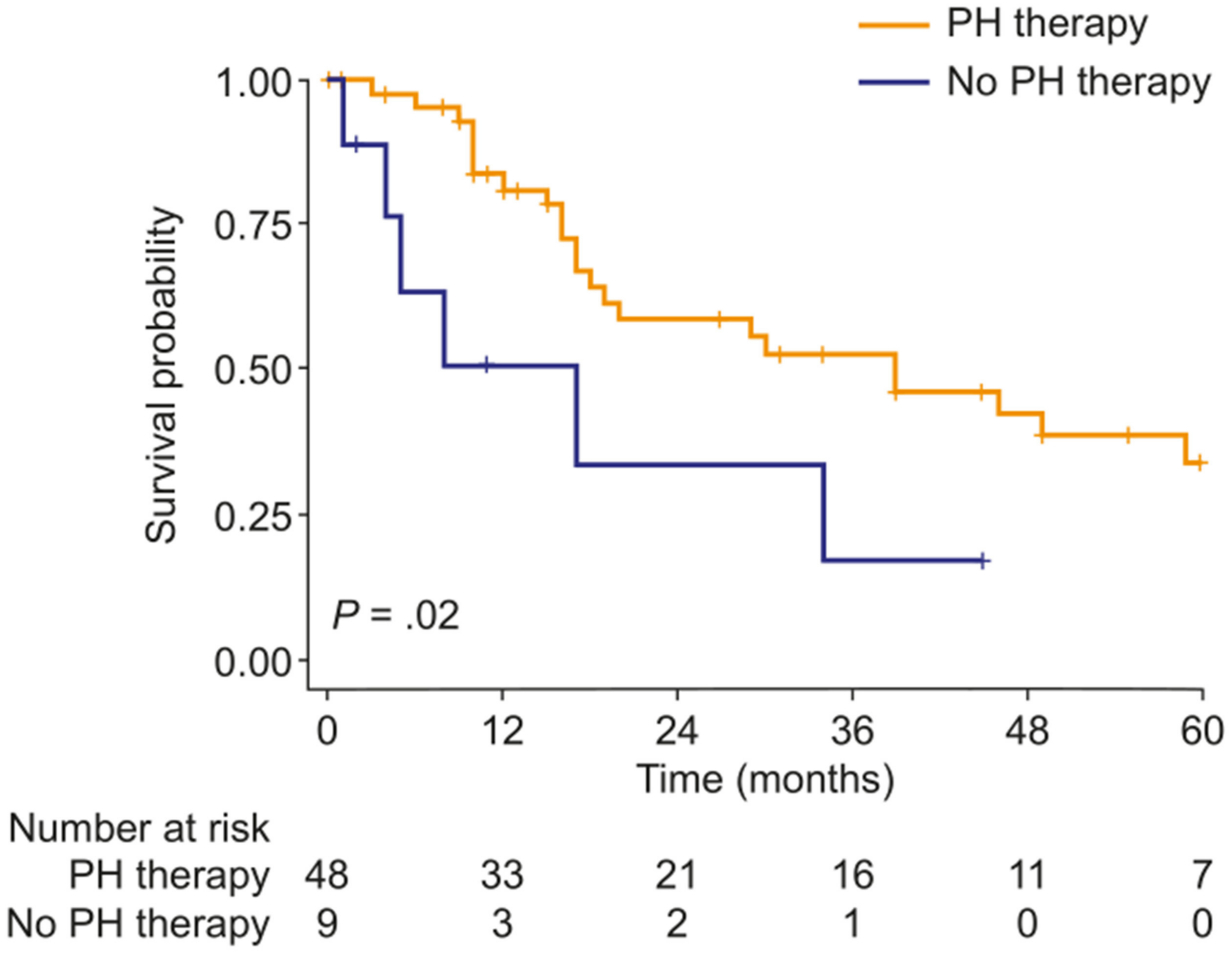
Kaplan–Meier curves of survival probability in patients with PH due to COPD with both TAPSE/PASP and 6MWD values in the lowest or intermediate tertile, stratified by use of targeted PH therapy. 6MWD, 6-min walk distance; PH, pulmonary hypertension; TAPSE/PASP, tricuspid annular plane systolic excursion/pulmonary artery systolic pressure.

Targeted PH therapies were initiated in 57 patients with mild-to-moderate PH-COPD and 93 patients with severe PH-COPD. In both groups, survival of patients with targeted PH therapy did not differ from survival of patients without targeted PH therapy (Figure 2). We next evaluated survival with vs. without targeted PH therapy in the three risk groups defined by TAPSE/PASP and 6MWD. Targeted PH therapies were taken by 10 of 26 patients at low risk, 32 of 48 patients at intermediate risk, and 41 of 49 patients at high risk. Survival with vs. without targeted PH therapy showed no significant difference in any of the three risk groups (Supplementary Figure 4). We also classified the patients according to TAPSE/PASP tertiles (<0.28 mm/mm Hg, 0.28–0.41 mm/mm Hg and >0.41 mm/mm Hg) and 6MWD tertiles (<200, 200–300, and >300 m). In the 63 patients who were in the lowest or intermediate tertiles of TAPSE/PASP and 6MWD, those who received targeted PH therapy (*n* = 50) had a higher survival rate than those without PH therapy (Figure 3; log-rank *p* = 0.02; 3-year survival: 53 and 17%, respectively; HR: 0.36; 95% CI: 0.14–0.89). Of the patients receiving targeted PH therapy, most received monotherapy (94%) with a phosphodiesterase 5 inhibitor (84%) or an endothelin receptor antagonist (16%).

## Discussion

The present results show that disease severity in COPD and survival in PH-COPD are predicted by cor pulmonale (assessed as RV-PA uncoupling and decreased exercise capacity) rather than severity of PH or risk scores derived from PAH research. Among patients with low TAPSE/PASP and 6MWD, those who received targeted PH therapies had better survival than those without PH therapies, although statistical significance depended on the TAPSE/PASP and 6MWD thresholds used.

The notion of a right-sided phenotype of heart failure in patients with chronic lung diseases is not new. In 1963, a World Health Organization-sponsored expert consensus conference reviewed chronic lung diseases-associated PH as a cause of heart failure, and defined “cor pulmonale” as RV hypertrophy and dilatation resulting from diseases affecting the structure or function of the lungs (6). This morphological definition proved impractical, and cor pulmonale became better understood as altered RV structure and function with eventual right heart failure symptomatology caused by PH on a background of pulmonary disease, most commonly COPD (7–9). It is interesting that echocardiographic signs of cor pulmonale may be found in patients with COPD and minimally increased PAP, suggesting that factors other than only PH alter RV-PA coupling in COPD (20).

The clinical assessment of cor pulmonale traditionally relied on radionuclide angiography for measurements of RV volumes and derived ejection fraction (EF). This approach established that RVEF is depressed and/or fails to increase during exercise in up to 50% of patients with advanced COPD, and may improve with supplemental oxygen or a variety of pulmonary vasodilating interventions including aminophylline, β2 stimulant drugs or nitrates (8, 9). Radionuclide RVEF was found in one study of 115 patients with COPD to be weakly but significantly correlated to survival (21). Radionuclide angiography has since been replaced by cardiac magnetic resonance imaging (22) or echocardiography (20) for the evaluation of cor pulmonale in COPD, but there has been no report of RVEF or any other measure of RV function as an independent predictor of outcome in patients with COPD.

In the present study, coupling of the right ventricle to afterload in COPD was assessed by simple 2D echocardiography. As recently reviewed, the right ventricle adapts to increased afterload by increasing contractility (23, 24). Therefore, correcting contractility (estimated by TAPSE) by an indirect measure of afterload (PASP) provides a more relevant assessment of RV function in patients with various forms of PH (10–14).

The present results confirm the importance of the TAPSE/PASP ratio in COPD both with and without PH. In COPD without PH, the TAPSE/PASP ratio correlated well with specific lung function parameters and 6MWD. Consistent with this finding, patients with a more advanced stage of COPD (mirrored by GOLD stages, mMRC score, need for oxygen supplementation, and presence of exacerbations leading to inpatient treatment) showed significantly reduced RV-PA coupling. In PH-COPD, the TAPSE/PASP ratio was an independent predictor of mortality, whereas the severity of PH alone was not prognostic. This may be a reason why all treatments that aim to decrease PVR or PAP have thus far failed to improve the prognosis of patients with PH-COPD (1).

The most recent proposed classification of severity of PH-COPD from the 6th World Symposium on PH attempts to identify those patients with advanced pulmonary vascular disease who might benefit from the cautious use of targeted therapies, preferably in controlled studies (1). Our patients with moderate or severe PH had a typical profile of high PAP but relatively limited alteration in lung function tests (2–4). It is therefore understandable that targeted PH therapies were prescribed for the treatment of significant pulmonary vascular disease or PAH co-morbidity (1). This strategy was not associated with a detectable effect on outcome when the patient population was analyzed as a whole. However, PH therapy was associated with increased survival in a subgroup analysis of patients in the lowest or intermediate tertiles of both TAPSE/PASP and 6MWD. These results suggest that future trials of targeted PH therapies in PH-COPD should recruit patients with more advanced RV-PA uncoupling, focusing on cor pulmonale rather than the severity of PH *per se*.

Exercise capacity is markedly reduced in COPD, in proportion to severity of the disease as assessed by the GOLD staging system (25). In a study of 365 patients with COPD, mortality was high (47% during a mean follow-up period of 5.5 years) and was predicted equally well by 6MWD and peak oxygen uptake (26). In another study of 362 patients with COPD who underwent cardiac catheterization and a 6MWD test as part of evaluation for lung transplantation, the prevalence of PH (mPAP >25 mm Hg and pulmonary arterial occlusion pressure <16 mm Hg) was 23% and 6MWD declined by 11 m for every 5 mm Hg rise in mPAP, but with borderline significance (*p* = 0.04) (27). A smaller study of 29 patients with advanced stable COPD showed no significant association between mPAP and exercise capacity (28). Decreased exercise capacity in COPD is mainly related to a ventilatory limitation (26, 28), but analysis of convective and diffusive oxygen transport mechanisms also disclosed a possible influence of cardiac output on skeletal muscle oxygen extraction (29). The present results show the relevance of RV-PA uncoupling in patients with PH-COPD; this could be a possible cause of the cardiac output limitation seen during exercise.

Recent studies have suggested that DLCO is an important predictor of mortality in patients with PH due to chronic lung disease (30, 31). However, univariate Cox regression analysis indicated that DLCO is not associated with 5-year survival in our study cohort. Although the underlying reason for the observed difference cannot be directly assessed in a retrospective cohort analysis, our study supports a role for DLCO as a predictor of short-term mortality.

There are several limitations to the present findings. The study was conducted at a single centre in Germany; the study population may therefore not be representative of the wider population with COPD. The generalizability of the results is also affected by referral bias, as the patients were evaluated at the request of their physicians for a suspicion of PH. Furthermore, only few patients in the high-risk group did not receive targeted PH therapies, and conclusions regarding the efficacy of PH therapies cannot be drawn from this retrospective cohort study. Nevertheless, the data draw attention to the relevance of cor pulmonale in patients with COPD, and support the enrichment of future clinical trial populations for patients with very low TAPSE/PASP and 6MWD.

Overall, we have provided evidence that cor pulmonale [assessed as RV-PA uncoupling (TAPSE/PASP ratio) and decreased exercise capacity (6MWD)] is associated with disease severity in COPD and prognosis in PH-COPD. Further studies are needed to assess the effect of targeted PH therapy in patients with PH-COPD and low TAPSE/PASP and 6MWD.

## Data Availability Statement

The raw data supporting the conclusions of this article will be made available by the authors, without undue reservation.

## Ethics Statement

The studies involving human participants were reviewed and approved by University of Giessen institutional review board (#266/11), Klinikstraße 29, 35392 Gießen. The patients/participants provided their written informed consent to participate in this study.

## Author Contributions

AY, MR, and KT conceived the idea for the analyses detailed in this manuscript. AY undertook statistical analyses of the data in the manuscript. All authors contributed to the design, data collection in the study, drafting and critical review of the manuscript, and approved the manuscript for submission.

## Funding

This study was funded by the Deutsche Forschungsgemeinschaft (DFG, German Research Foundation) – Projektnummer 268555672 – SFB 1213, Project B08. Editorial assistance was provided by Claire Mulligan, PhD (Beacon Medical Communications Ltd, Brighton, UK), funded by the University of Giessen.

## Conflict of Interest

AY reports non-financial support from the University of Giessen during the conduct of the study. SK reports personal fees from Chiesi, Berlin Chemie MENARINI and Insmed, and personal fees and non-financial support from GSK, Novartis and AstraZeneca outside the submitted work. HG reports grants from the German Research Foundation and non-financial support from the University of Giessen during the conduct of the study, and personal fees from Actelion, AstraZeneca, Bayer, BMS, GSK, Janssen-Cilag, Lilly, MSD, Novartis, OMT, Pfizer and United Therapeutics outside the submitted work. MF reports non-financial support from the University of Giessen during the conduct of the study. HG reports grants from the German Research Foundation and nonfinancial support from the University of Giessen during the conduct of the study, and personal fees from Bayer, Actelion, Pfizer, Merck, GSK and Takeda, grants and personal fees from Novartis, Bayer HealthCare and Encysive/Pfizer, and grants from Aires, the German Research Foundation, Excellence Cluster Cardiopulmonary Research and the German Ministry for Education and Research outside the submitted work. RN reports relationships including consultancies, speaker's fees and membership of advisory boards with AOP Orphan Pharmaceuticals, Johnson & Johnson, Lung Biotechnology Corporation and United Therapeutics. WS reports grants from the German Research Foundation and nonfinancial support from the University of Giessen during the conduct of the study, and personal fees from Pfizer and Bayer Pharma AG outside the submitted work. MR reports grants from the German Research Foundation and non-financial support from the University of Giessen during the conduct of the study, and grants from United Therapeutics, grants and personal fees from Bayer, and personal fees from Actelion, Mundipharma, Roche and OMT outside the submitted work. KT reports grants from the German Research Foundation and non-financial support from the University of Giessen during the conduct of the study, and personal fees from Actelion and Bayer outside the submitted work. The remaining authors declare that the research was conducted in the absence of any commercial or financial relationships that could be construed as a potential conflict of interest.

## Publisher's Note

All claims expressed in this article are solely those of the authors and do not necessarily represent those of their affiliated organizations, or those of the publisher, the editors and the reviewers. Any product that may be evaluated in this article, or claim that may be made by its manufacturer, is not guaranteed or endorsed by the publisher.

## References

[1] Nathan SD Barbera JA Gaine SP Harari S Martinez FJ Olschewski H. Pulmonary hypertension in chronic lung disease and hypoxia. Eur Respir J. (2019) 53:1801914. 10.1183/13993003.01914-2018 30545980 PMC6351338

[2] Andersen KH Iversen M Kjaergaard J Mortensen J Nielsen-Kudsk JE Bendstrup E. Prevalence, predictors, and survival in pulmonary hypertension related to end-stage chronic obstructive pulmonary disease. J Heart Lung Transplant. (2012) 31:373–80. 10.1016/j.healun.2011.11.020 22226804

[3] Chaouat A Bugnet AS Kadaoui N Schott R Enache I Ducolone A. Severe pulmonary hypertension and chronic obstructive pulmonary disease. Am J Respir Crit Care Med. (2005) 172:189–94. 10.1164/rccm.200401-006OC 15831842

[4] Thabut G Dauriat G Stern JB Logeart D Levy A Marrash-Chahla R. Pulmonary hemodynamics in advanced COPD candidates for lung volume reduction surgery or lung transplantation. Chest. (2005) 127:1531–6. 10.1378/chest.127.5.1531 15888824

[5] Bishop JM. Hypoxia and pulmonary hypertension in chronic bronchitis. Prog Respir Res. (1975) 9:10–6. 10.1159/00039815817584724

[6] Dankmeijer J Herles F Ibrahim M Reid DD Richards DW Stuart- Harris CH. Chronic Cor Pulmonale. World health organization technical report series No. 213. Circulation. (1963) 27:594–615. 10.1161/01.CIR.27.4.59414447583

[7] Fishman AP. State of the art: chronic cor pulmonale. Am Rev Respir Dis. (1976) 114:775–94. 10.1164/arrd.1976.114.4.775 788575

[8] MacNee W. Pathophysiology of cor pulmonale in chronic obstructive pulmonary disease. Part One. Am J Respir Crit Care Med. (1994) 150:833–52. 10.1164/ajrccm.150.3.8087359 8087359

[9] MacNee W. Pathophysiology of cor pulmonale in chronic obstructive pulmonary disease. Part two. Am J Respir Crit Care Med. (1994) 150:1158–68. 10.1164/ajrccm.150.4.7921453 7921453

[10] Guazzi M Dixon D Labate V Beussink-Nelson L Bandera F Cuttica MJ. RV contractile function and its coupling to pulmonary circulation in heart failure with preserved ejection fraction: stratification of clinical phenotypes and outcomes. JACC Cardiovasc Imag. (2017) 10:1211–21. 10.1016/j.jcmg.2016.12.024 28412423

[11] Tello K Wan J Dalmer A Vanderpool R Ghofrani HA Naeije R. Validation of the tricuspid annular plane systolic excursion/systolic pulmonary artery pressure ratio for the assessment of right ventricular-arterial coupling in severe pulmonary hypertension. Circ Cardiovasc Imag. (2019) 12:e009047. 10.1161/CIRCIMAGING.119.009047 31500448 PMC7099862

[12] Tello K Ghofrani HA Heinze C Krueger K Naeije R Raubach C. A simple echocardiographic estimate of right ventricular-arterial coupling to assess severity and outcome in pulmonary hypertension on chronic lung disease. Eur Respir J. (2019) 54:1802435. 10.1183/13993003.02435-2018 31073085

[13] Guazzi M Bandera F Pelissero G Castelvecchio S Menicanti L Ghio S. Tricuspid annular plane systolic excursion and pulmonary arterial systolic pressure relationship in heart failure: an index of right ventricular contractile function and prognosis. Am J Physiol Heart Circ Physiol. (2013) 305:H1373–81. 10.1152/ajpheart.00157.2013 23997100

[14] Tello K Axmann J Ghofrani HA Naeije R Narcin N Rieth A. Relevance of the TAPSE/PASP ratio in pulmonary arterial hypertension. Int J Cardiol. (2018) 266:229–35. 10.1016/j.ijcard.2018.01.053 29887454

[15] Galie N Humbert M Vachiery JL Gibbs S Lang I Torbicki A. 2015 ESC/ERS Guidelines for the diagnosis and treatment of pulmonary hypertension: the joint task force for the diagnosis and treatment of pulmonary hypertension of the European society of cardiology (ESC) and the European respiratory society (ERS): endorsed by: association for European paediatric and congenital cardiology (AEPC), international society for heart and lung transplantation (ISHLT). Eur Respir J. (2015) 46:903–75. 10.1183/13993003.01032-2015 26318161

[16] Yogeswaran A Tello K Faber M Sommer N Kuhnert S Seeger W. Risk assessment in severe pulmonary hypertension due to interstitial lung disease. J Heart Lung Transplant. (2020) 39:1118–25. 10.1016/j.healun.2020.06.014 32690230

[17] Gall H Felix JF Schneck FK Milger K Sommer N Voswinckel R. The giessen pulmonary hypertension registry: survival in pulmonary hypertension subgroups. J Heart Lung Transplant. (2017) 36:957–67. 10.1016/j.healun.2017.02.016 28302503

[18] Yogeswaran A Richter MJ Sommer N Ghofrani HA Seeger W Gall H. Evaluation of pulmonary hypertension by right heart catheterisation: does timing matter? Eur Respir J. (2020) 56:1901892. 10.1183/13993003.01892-2019 32350102

[19] Hoeper MM Kramer T Pan Z Eichstaedt CA Spiesshoefer J Benjamin N. Mortality in pulmonary arterial hypertension: prediction by the 2015 European pulmonary hypertension guidelines risk stratification model. Eur Respir J. (2017) 50:1700740. 10.1183/13993003.00740-2017 28775047

[20] Hilde JM Skjorten I Grotta OJ Hansteen V Melsom MN Hisdal J. Right ventricular dysfunction and remodeling in chronic obstructive pulmonary disease without pulmonary hypertension. J Am Coll Cardiol. (2013) 62:1103–11. 10.1016/j.jacc.2013.04.091 23831444

[21] France AJ Prescott RJ Biernacki W Muir AL MacNee W. Does right ventricular function predict survival in patients with chronic obstructive lung disease? Thorax. (1988) 43:621–6. 10.1136/thx.43.8.621 3175974 PMC461398

[22] Gao Y Du X Qin W Li K. Assessment of the right ventricular function in patients with chronic obstructive pulmonary disease using MRI. Acta Radiol. (2011) 52:711–5. 10.1258/ar.2011.100449 21852436

[23] Sanz J Sanchez-Quintana D Bossone E Bogaard HJ Naeije R. Anatomy, function, and dysfunction of the right ventricle: JACC state-of-the-art review. J Am Coll Cardiol. (2019) 73:1463–82. 10.1016/j.jacc.2018.12.076 30922478

[24] Vonk Noordegraaf A Chin KM Haddad F Hassoun PM Hemnes AR Hopkins SR. Pathophysiology of the right ventricle and of the pulmonary circulation in pulmonary hypertension: an update. Eur Respir J. (2019) 53:1801900. 10.1183/13993003.01900-2018 30545976 PMC6351344

[25] Pinto-Plata VM Celli-Cruz RA Vassaux C Torre-Bouscoulet L Mendes A Rassulo J. Differences in cardiopulmonary exercise test results by American thoracic society/European respiratory society-global initiative for chronic obstructive lung disease stage categories and gender. Chest. (2007) 132:1204–11. 10.1378/chest.07-0593 17934113

[26] Cote CG Pinto-Plata V Kasprzyk K Dordelly LJ Celli BR. The 6-min walk distance, peak oxygen uptake, and mortality in COPD. Chest. (2007) 132:1778–85. 10.1378/chest.07-2050 17925409

[27] Sims MW Margolis DJ Localio AR Panettieri RA Kawut SM Christie JD. Impact of pulmonary artery pressure on exercise function in severe COPD. Chest. (2009) 136:412–9. 10.1378/chest.08-2739 19318664 PMC2818413

[28] Pynnaert C Lamotte M Naeije R. Aerobic exercise capacity in COPD patients with and without pulmonary hypertension. Respir Med. (2010) 104:121–6. 10.1016/j.rmed.2009.06.006 19577458

[29] Broxterman RM Hoff J Wagner PD Richardson RS. Determinants of the diminished exercise capacity in patients with chronic obstructive pulmonary disease: looking beyond the lungs. J Physiol. (2020) 598:599–610. 10.1113/JP279135 31856306 PMC6995414

[30] Balasubramanian A Kolb TM Damico RL Hassoun PM McCormack MC Mathai SC. Diffusing capacity is an independent predictor of outcomes in pulmonary hypertension associated with COPD. Chest. (2020) 158:722–34. 10.1016/j.chest.2020.02.047 32184109 PMC8173778

[31] Rose L Prins KW Archer SL Pritzker M Weir EK Misialek JR. Survival in pulmonary hypertension due to chronic lung disease: influence of low diffusion capacity of the lungs for carbon monoxide. J Heart Lung Transplant. (2019) 38:145–55. 10.1016/j.healun.2018.09.011 30391191 PMC6556403

