# Supplementary material for: Relevance of Cor Pulmonale in COPD With and Without Pulmonary Hypertension: A Retrospective Cohort Study
Source: Front Cardiovasc Med. 2022 Feb 16;9:826369. doi: 10.3389/fcvm.2022.826369 (PMC8889008; doi:10.3389/fcvm.2022.826369)
Supplement: Supplementary file 1 [file Data_Sheet_1.PDF]

1 Supplementary Figures

(A)

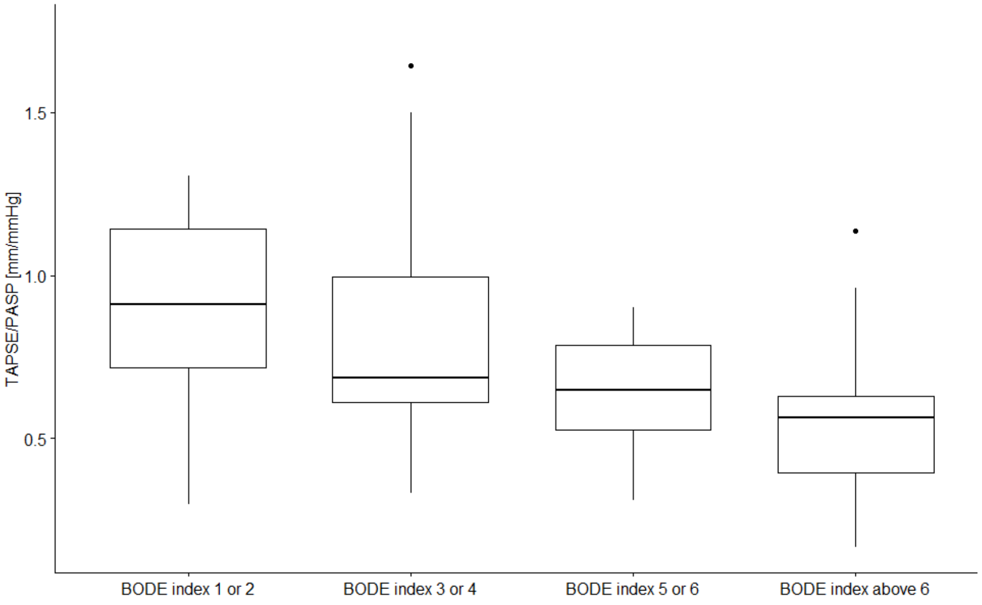

(B)

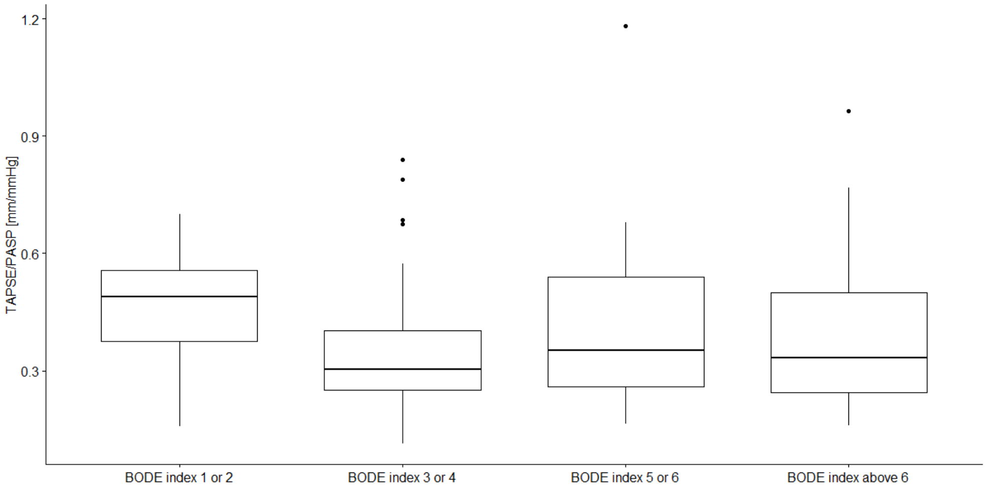

SUPPLEMENTARY FIGURE S1 | TAPSE/PASP ratio stratified by BODE index in **(A)** patients with COPD without PH (Kruskal–Wallis $p = 0.034$; $n = 51$) and **(B)** patients with PH due to COPD (Kruskal–Wallis $p = 0.50$; $n = 85$). Medians, IQRs, maximum and minimum values (within 1.5x the IQR from the upper and lower quartiles) and outliers are shown. BODE, body mass index, obstruction, dyspnoea and exercise capacity; COPD, chronic obstructive pulmonary disease; IQR, interquartile range; PH, pulmonary hypertension; TAPSE/PASP, tricuspid annular plane systolic excursion/pulmonary artery systolic pressure.

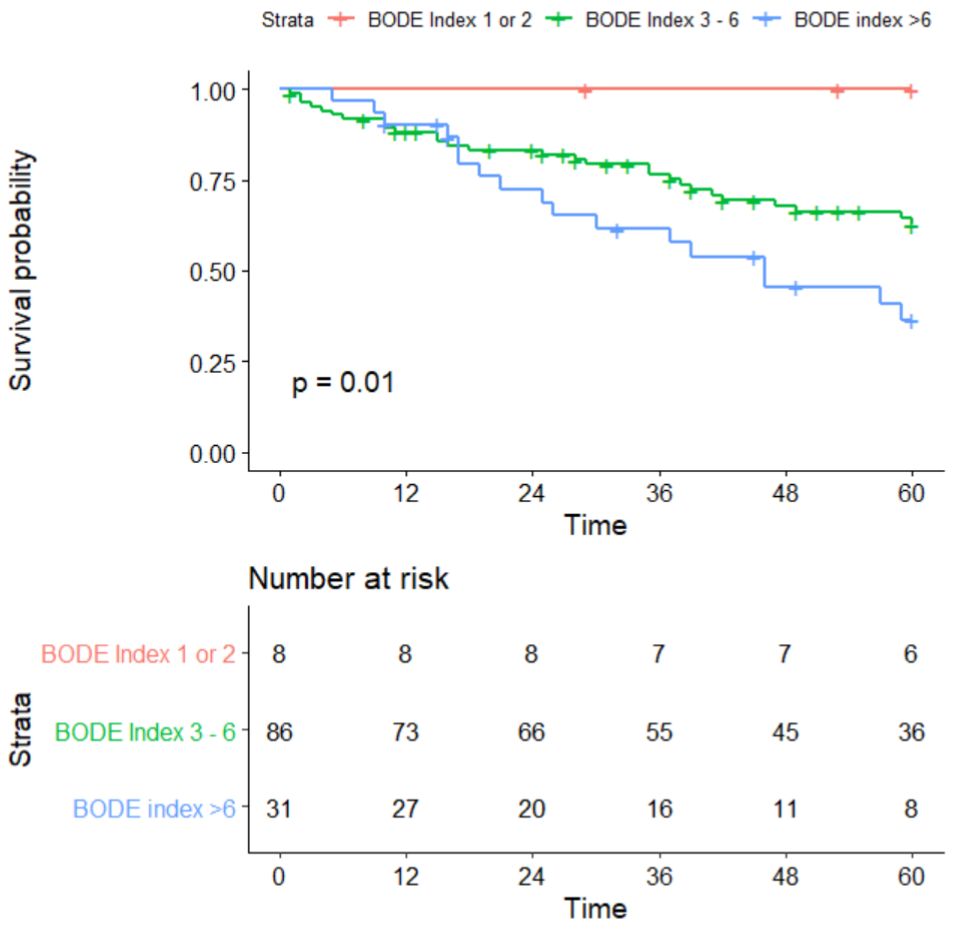

SUPPLEMENTARY FIGURE S2 | Kaplan–Meier curves of survival probability in patients with pulmonary hypertension due to chronic obstructive pulmonary disease stratified by BODE index. BODE, body mass index, obstruction, dyspnoea and exercise capacity.

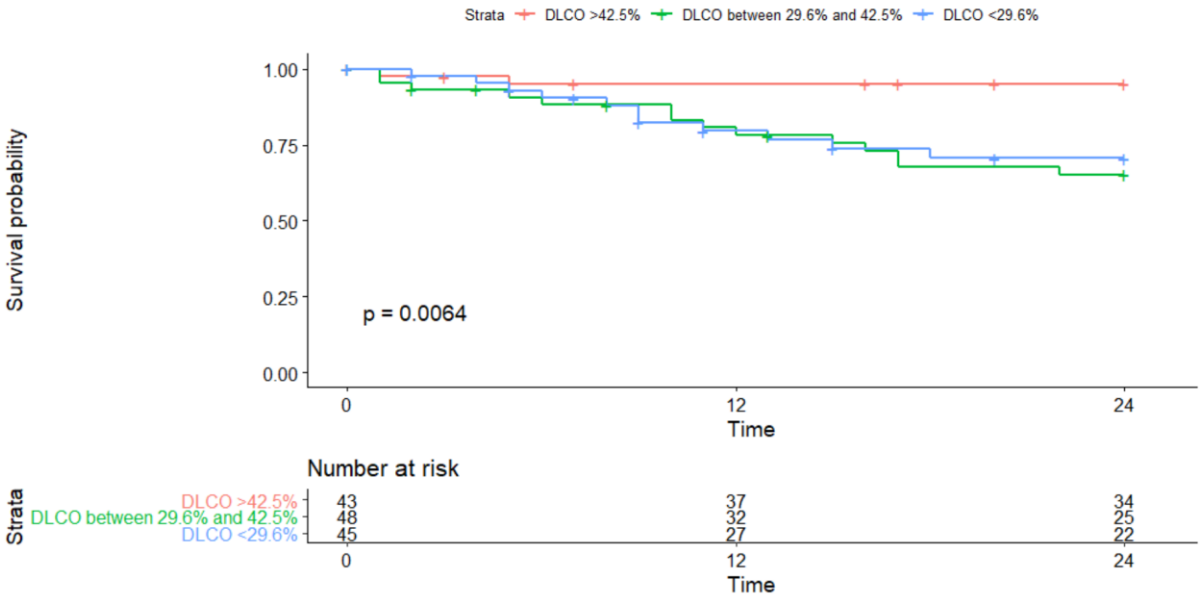

SUPPLEMENTARY FIGURE S3 | Kaplan–Meier curves of survival probability in patients with pulmonary hypertension due to chronic obstructive pulmonary disease stratified by DLCO tertile. A significant difference in short-term survival (truncated at 2 years) was observed. Consistent with this finding, Cox regression analysis showed a decreased risk of short-term mortality with increasing DLCO (hazard ratio: 0.968 [95% confidence interval: 0.943–0.994]; $p = 0.0159$). DLCO, lung diffusing capacity for carbon monoxide.

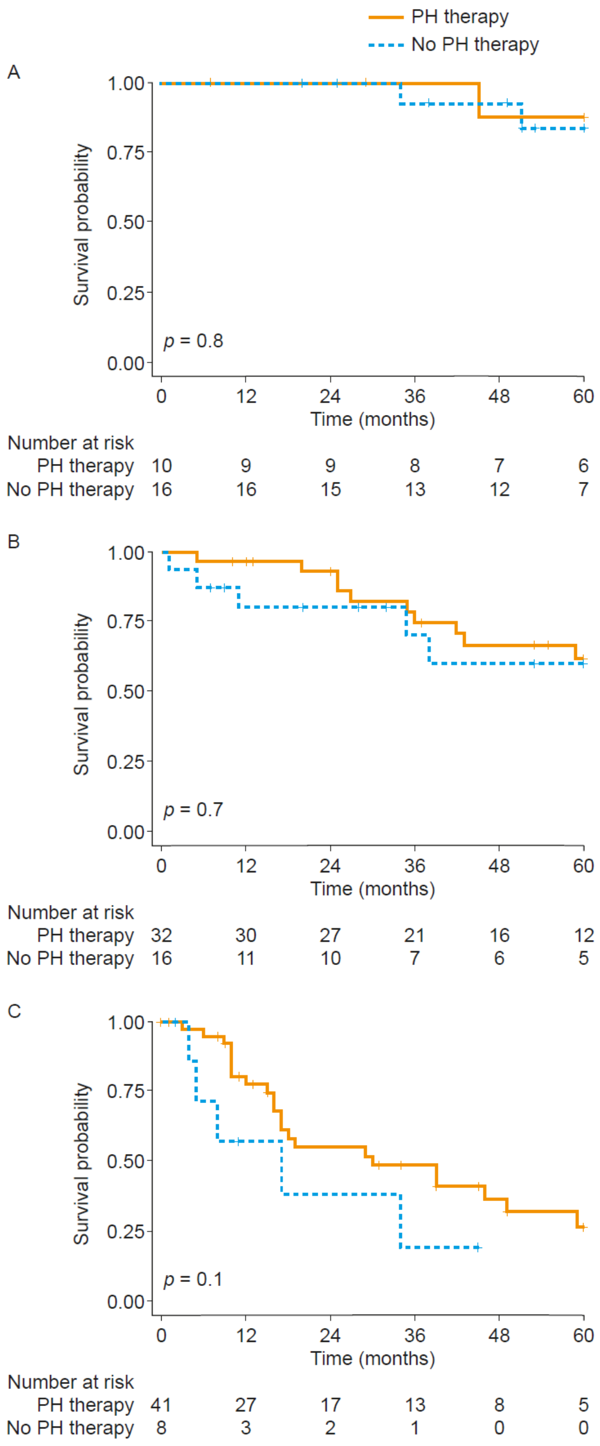

SUPPLEMENTARY FIGURE S4 | Kaplan–Meier curves of survival probability in patients with PH due to COPD and **(A)** TAPSE/PASP > 0.35 mm/mm Hg and 6MWD > 299 m (low risk), **(B)** TAPSE/PASP > 0.35 mm/mm Hg or 6MWD > 299 m (intermediate risk) or **(C)** TAPSE/PASP ≤ 0.35 mm/mm Hg and 6MWD ≤ 299 m (high risk), stratified by use of targeted PH therapy. 6MWD, 6-minute walk distance; PH, pulmonary hypertension; TAPSE/PASP, tricuspid annular plane systolic excursion/pulmonary artery systolic pressure.

2 Supplementary Table

SUPPLEMENTARY TABLE S1 | Correlation of the TAPSE/PASP ratio with haemodynamic parameters in patients with PH-COPD.

**Parameter**	**Pearson R**	***p***
Mean pulmonary artery pressure	−0.425	<0.001
Right atrial pressure	−0.072	0.344
Cardiac Index	0.282	<0.001
Mixed venous oxygen saturation	0.398	<0.001

PH-COPD, pulmonary hypertension due to chronic obstructive pulmonary disease; TAPSE/PASP, tricuspid annular plane systolic excursion/pulmonary artery systolic pressure.
